# Differences between the antibiotic prescribing pattern of newly arrived refugees in Germany and the German population

**DOI:** 10.1186/s13031-018-0139-z

**Published:** 2018-01-29

**Authors:** Fabian Kahl, Thomas Kühlein

**Affiliations:** 1 Institut für Geschichte und Ethik der Medizin, Friedrich-Alexander Universität Erlangen-Nürnberg, Glückstraße 10, 91054 Erlangen, Germany; 2 Allgemeinmedizinisches Institut, Friedrich-Alexander Universität Erlangen-Nürnberg, Universitätsstraße 29, 91054 Erlangen, Germany

## Abstract

The number of refugees arriving in Europe increased dramatically in 2015, challenging the German health system. Amongst others, the treatment of infectious diseases is an important topic in refugee healthcare. A high prevalence of multi-drug-resistant organisms has been identified among the refugee population. Still, little is known about the prescription of antibiotic medication for refugees. We conducted a descriptive analysis of all antibiotics prescribed to newly arrived refugees who were treated as outpatients between 10/01/2014 and 09/30/2015 in Erlangen, an average sized German town. The City’s invoicing documents were used to collect data on prescriptions written for refugees. Basic penicillins, aminopenicillins with beta-lactamase inhibitor and cephalosporins constituted the largest proportion of antibiotics prescribed in the adult refugee group. Of these, both aminopenicillins with beta-lactamase-inhibitor as well as basic penicillins were prescribed significantly more often compared to non-refugees. We conclude that the high percentage of prescriptions of aminopenicillins with beta-lactamase inhibitor is striking and should be further investigated.

## Correspondence

Since the beginning of the refugee crisis in 2015, medical care for refugees has posed a challenge to European healthcare systems, including the German health system. Amongst many other health problems these patients present with, infectious diseases are salient [1, 2], including a high prevalence of multidrug-resistant organisms (MDRO) like methicillin-resistant *Staphylococcus aureus* (MRSA) [3–6]. Little is known about the medical treatment of infectious diseases of refugees in Germany [7], but the studies mentioned above call for a better surveillance of prescribed antibiotics, as they can be both a cause and a consequence of a high MDRO prevalence. Therefore, we present here the pattern of antibiotics prescribed to newly arrived refugees in the city of Erlangen, assessed through a retrospective analysis of all outpatient prescriptions for newly arrived refugees in Erlangen over the period from 10/01/2014–09/30/2015. The data basis we used were anonymised invoicing documents of the City’s Department of Social Services.

For a better comparison with literature reporting on antibiotic prescriptions in Germany, we calculated the defined daily dose (DDD) [8] of each antibiotic prescribed and classified the substances in classes according to the classification of Augustin et al. [9], as also used by Batzing-Feigenbaum et al. [10]. Table 1 shows all antibiotic substances prescribed in this study as classes.

**Table 1:** Overview and Classification of all prescribed antibiotics

Antibiotic group	ATC-classes	Substance
Aminopenicillins with beta-lactamase inhibitor (BLI)	J01CR02	amoxicillin and enzyme inhibitor
	J01CR04	sultamicillin
Basic penicillins	J01CA04	amoxicillin
	J01CE02	phenoxymethylpenicillin
	J01CE10	benzathine phenoxymethylpenicillin
Cephalosporins	J01DC02	cefuroxime
	J01DC04	cefaclor
	J01DD08	cefixime
	J01DD13	cefpodoxime
	J01DD14	ceftibuten
Fluoroquinolones	J01MA01	ofloxacin
	J01MA02	ciprofloxacin
	J01MA12	levofloxacin
Macrolides/lincosamides	J01FA01	erythromycin
	J01FA06	roxithromycin
	J01FA09	clarithromycin
	J01FA10	azithromycin
	J01FF01	clindamycin
Nitrofurantoin/fosfomycin/nitroxolin	J01XE01	nitrofurantoin
	J01XX01	fosfomycin
Sulfonamide/trimethoprim	J01EE01	sulfamethoxazole and trimethoprim
Tetracyclines	J01AA02	doxycycline

We analysed the antibiotic prescribing pattern of two groups which, due to their size, allowed a meaningful analysis: Persons from 0 to 14 years, in the following referred to as “children group”, and persons from 20 to 39 years, in the following referred to as “adult group”.

While we did not find major differences in the children group (0–14 years) compared to children insured by the German statutory health insurance (gesetzliche Krankenversicherung, GKV), as our results (see Table 2) are equal to the analyses of GKV-insured children by Augustin et al. [9] and Bätzing-Feigenbaum et al. [10], we found striking deviations in the adult group: Here, of 478 drugs prescribed in total, 100 were antibiotics. Antibiotics therefore accounted for 20.9% of all prescriptions in this group. The most frequently prescribed class of antibiotics was basic penicillins (31%), followed by aminopenicillins with beta-lactamase inhibitors (BLI) (21%) and cephalosporins (16%).

**Table 2:** Prescriptions of antibiotic classes in the adult and children group

Prescriptions in DDD	adults (20–39 years)		children (0–14 years)	
Antibiotic groups	DDD absolute	DDD relative	DDD absolute	DDD relative
Aminopenicillins with beta-lactamase inhibitor	218,5	20,62%	10,50	2,61%
Basic penicillins	466,5	44,03%	200,50	49,81%
Cephalosporins	141,5	13,36%	110,50	27,45%
Fluoroquinolones	21	1,98%	0	0,00%
Macrolides/lincosamides	109	10,29%	55,00	13,66%
Metronidazol	0	0,00%	0	0,00%
Nitrofurantoin/fosfomycin/nitroxolin	28	2,64%	1,00	0,25%
Sulfonamide/trimethoprim	35	3,30%	5,00	1,24%
Tetracyclines	40	3,78%	20,00	4,97%
Others	0	0,00%	0	0,00%
Total	1059,5	100,00%	402,50	100,00%

To adults, overall 1059.5 DDD of antibiotics were prescribed. Basic penicillins constituted the greatest part with 44%, followed by aminopenicillins with BLI (21%), cephalosporins (13%) and macrolides (10%) (see Table 2).

In comparison to GKV-insured patients, refugees received broad spectrum antibiotics and aminopenicillins with BLI more often. Bätzing-Feigenbaum et al. [10] found a percentage of 25.9% for basic penicillins of all prescribed antibiotic DDD, while aminopenicillins with BLI had a rate of 2.9% in the age group 15–69 years [10]. Another striking difference is found for the prescriptions of tetracyclines, where we found a percentage of 3.8% at all prescribed DDD in the adult group, while for the GKV-insured persons, Bätzing-Feigenbaum et al. observed a percentage of 17.7% for tetracyclines [10]. Unfortunately, when analysing the prescriptions, it was not possible to infer anything about the diagnosis leading to the prescriptions. Therefore, a specific analysis as to the cause of the strikingly high percentage of aminopenicillins with BLI in the adult group could not be carried out. One could assume that the illnesses treated in the refugee group were dissimilar to the GKV-insured. However, this seems not to be the case. Alberer et al. [2] and Jung [1] described the distribution of diagnoses in refugee patients. Beside symptom diagnoses, respiratory tract infections were the most common. It is well known that these diagnoses are typical for almost any GP’s office, so the spectrum of diseases seems unlikely to account for the deviant antibiotic prescription.

Unfortunately, we can only report on a small number of antibiotic prescriptions. It was also not possible to gain information about the diagnoses leading to the prescriptions. However, we offer the first insight into the antibiotic prescription pattern of refugees in Germany.

## Conclusion

Due to the fact, that the prevalence for MRSA in refugees is not only higher compared to the German population [4], but exceeds the prevalence of MRSA in other known risk groups [5], the high prescription rate of aminopenicillins with BLI in the adult group might be reason for alarm. Considering the limitations of the study, we see that data regarding refugee health is lacking and a better surveillance of prescription rates is required.
